# Exploring the bidirectional associations between loneliness and cognitive functioning over 10 years: the English longitudinal study of ageing

**DOI:** 10.1093/ije/dyz085

**Published:** 2019-05-05

**Authors:** Jiamin Yin, Camille Lassale, Andrew Steptoe, Dorina Cadar

**Affiliations:** 1 Department of Epidemiology and Public Health, London, UK; 2 Department of Behavioural Science in Health, University College London, London, UK

**Keywords:** Loneliness, memory, verbal fluency, cognitive decline, older people, bivariate dual change score models

## Abstract

**Background:**

As the population ages, cognitive decline and dementia have become major health concerns in the UK. Loneliness has been linked to cognitive decline, but the reverse causality of this association remains unclear. This study aims to examine whether there is a bidirectional relationship between loneliness and cognitive function in older English adults (age 50 years and over) over a 10-year follow-up.

**Methods:**

Data came from a nationally representative sample of 5885 participants in the English Longitudinal Study of Ageing (ELSA), free of stroke or dementia and followed every 2 years up to wave 7 (2014–15). At each wave, cognitive function was measured with word recall and verbal fluency tests, and loneliness was measured with the abridged version of the revised UCLA Loneliness Scale. Bivariate dual change score models were used to assess the multivariate associations between loneliness and cognitive function, used interchangeably as exposures and outcomes.

**Results:**

Greater loneliness at baseline was associated with poorer memory [*β intercept* = −0.03, standard error (SE) = 0.01, *P*  =  0.016] and verbal fluency (*β intercept* = −0.01, SE  =  001, *P* =  0.027) at baseline, and with a stronger linear rate of decline in both memory (*β linear slope =* −0.07, SE  =  001, *P*  ≤ 0.001) and verbal fluency (*β linear slope* = −0.09, SE  =  0.03, *P* =  0.003) over a 10-year follow-up period, although the performance on verbal fluency did not change substantially on average over this period. We also found that higher baseline memory, but not verbal fluency, predicted a slower change in loneliness (*β linear slope* = −0.01, SE  =  001, *P* =  0.004) and that a linear decline in memory was associated with an acceleration in loneliness (*β quadratic slope* = −0.02, SE  =  001, *P*  ≤ 0.001) during follow-up.

**Conclusions:**

Higher loneliness is associated with poorer cognitive function at baseline and contributes to a worsening in memory and verbal fluency over a decade. These factors seem, however, to be partially intertwined, since baseline memory and its rate of decline also contribute to an increase in loneliness over time.

**Key Messages**

- There is a cross-sectional association between baseline loneliness and cognitive functioning (memory and verbal fluency).
- Baseline loneliness predicts changes in both memory and verbal fluency over time, but only baseline memory predicts a change in loneliness over time. Although loneliness and depression appear closely linked, loneliness may, by itself and independently of depressive symptoms, be associated with memory decline over a 10-year follow-up period.
- Interventions to reduce accelerations in cognitive decline in older adults might usefully focus on alleviating loneliness and interrupting the possible vicious cycle between loneliness and cognitive deterioration.

## Introduction

Life expectancy in the UK is increasing rapidly.^1^ The male life expectancy was projected to increase from 89 years for an individual born in 2007 to 91 years for a man born in 2030 and, if female, the corresponding figures are 92 years in 2007 and 95 in 2030.^2^ However, increased life expectancy is not necessarily equivalent to healthy extra years of life,^2^ meaning that population ageing and age-related health problems are increasingly becoming a public health priority.^3^

Brain ageing leads to a decline in cognitive function, which is a slow, gradual process over time.^4^ Considerable declines in cognitive performance, including memory, verbal fluency and processing speed, can be seen among most older adults, starting from as early as midlife^5–9^; although other aspects of cognitive functioning, such as vocabulary or mastery, remain preserved until later in life.

Loneliness is a complex emotional state where perceptions of the adequacy of social contacts or the intimacy of the individual’s relationships are below the desired level.^10^ About 9% of British people aged 65 years and older experience loneliness.^11^^,^^12^

There is a growing number of studies exploring the association between loneliness and cognitive function in older adults, but these findings are relatively mixed^13^ and only a few studies to date have examined loneliness as a predictor of cognitive decline. Some of this evidence showed that loneliness was found to be associated with a poorer global cognitive function^14^^,^^15^ or related to specific measures of cognitive function, in particular verbal fluency^16^ or performance on a task of memory delayed recall.^17^ Previous work conducted in the English Longitudinal Study of Ageing (ELSA) investigated the association between loneliness at wave 2 and cognitive function at waves 2 and 4, finding a reverse cross-sectional association with poorer memory and verbal fluency at wave 2 but not with cognitive performance 4 years later, at wave 4.^18^

Loneliness was also linked with an accelerated cognitive decline over time, an association which was independent of psychological symptoms such as depressive symptomatology.^14^^,^^19^ This is in contrast to the work of Gow and colleagues, who have argued that depressive symptoms explain much of the association between cognitive function and loneliness.^20^

To date, only a limited number of studies have examined the bidirectional relationships between loneliness and cognitive function. An investigation based on data from the Health and Retirement Survey (HRS) 1998–2010 showed that greater loneliness at baseline predicted faster memory decline, but not vice versa.^21^ However, in another HRS investigation conducted from 2004 to 2012, it was observed that poorer memory had an adverse effect on loneliness, whereas loneliness did not predict memory decline.^22^ This was not the case for the analyses conducted in a large cohort of Chinese older adults,^23^ which showed that loneliness was associated with an accelerated cognitive decline over time, and poorer cognitive function predicted deterioration in loneliness.

In this study, we aimed to investigate loneliness not only as a predictor of memory and verbal fluency but also as a consequence of these cognitive abilities over a 10-year follow-up period, with repeated measurements every 2 years. This parallel analysis can offer a deeper understanding of the dual changes in cognitive function and loneliness over time, as well as potential reverse causation, while testing for a bidirectional association. Understanding the interplay between loneliness and cognitive function could have important implications for the early identification of cognitive decline and the role of loneliness as a modifiable risk factor.

Ethics approval for each one of the ELSA waves was granted by the National Research Ethics Service [London Multicenter Research Ethics Committee (MREC/01/2/91)] at [http://www.nres.npsa.nhs.uk]. All participants provided informed consent.

## Methods

### Data

**Figure 1.**
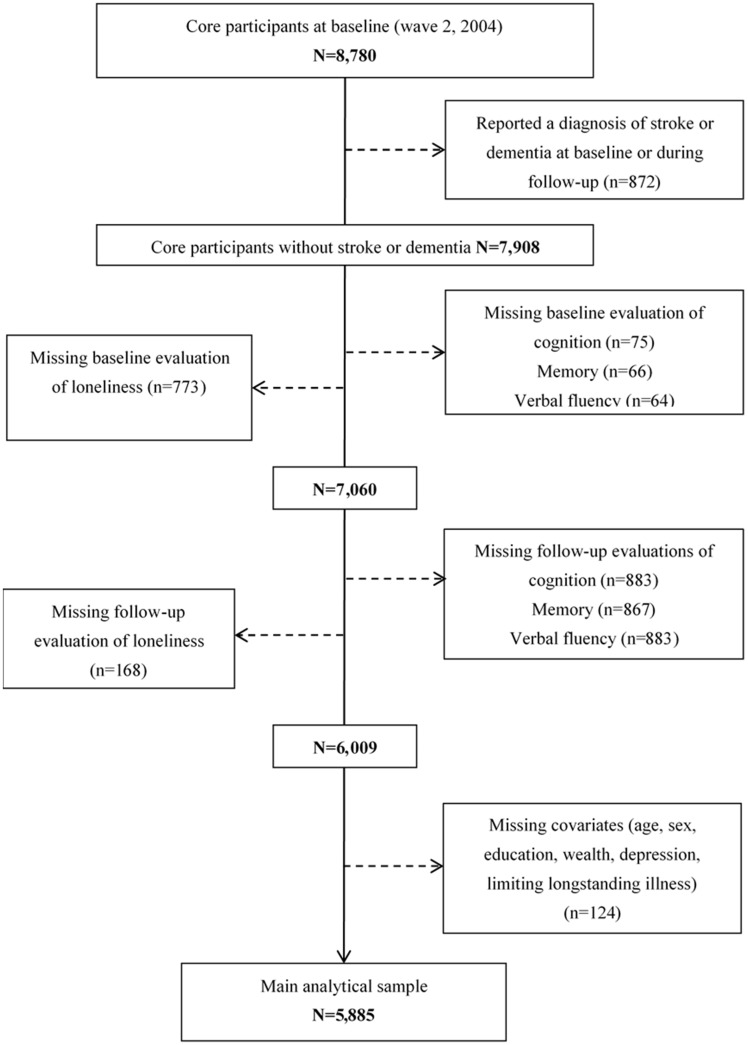
Flowchart representing the selection criteria of the analytical sample in ELSA.

ELSA is a panel study of a nationally representative sample of the English population living in private households and aged 50 years or older,^24^ which has been described in more detail elsewhere.^25^^,^^26^ Wave 2 (2004–05) was considered the baseline as it was the first wave in which a measure of loneliness was included in ELSA. For these analyses, data were available up to wave 7 (2014–15), constituting up to 10 years of follow-up. There were 8780 core members interviewed at baseline. Those who reported a diagnosis of stroke or dementia at baseline or during the follow-up period were excluded (*n*  =  872). Dementia occurrence was determined at each wave, using an algorithm based on a combination of self- or informant-reported physician diagnosis of dementia or Alzheimer disease or an informant-reported score above the threshold of 3.38 on the 16-question Informant Questionnaire on Cognitive Decline in the Elderly (IQCODE).^27^^,^^28^ For individuals with a self-reported physician diagnosis of stroke, we allowed the cognitive data before the wave where a stroke diagnosis had been confirmed and excluded the observations after the stroke incidence. We also excluded those who had no baseline or follow-up evaluation of cognitive function or loneliness or had missing covariates, leaving 5885 individuals in this analysis. The process of the analytical sample selection is shown in Figure 1.

### Cognitive function

Memory and verbal fluency were included in the cognitive assessment administered at each wave. Memory was assessed with a word recall test. A list of 10 words was assigned to every participant randomly. Participants were then asked to try to recall as many words as they could, both immediately and after a short delay. The numbers of words recalled correctly in immediate recall and delayed recall were combined and used as a continuous measure of memory, leading to a possible score ranging from 0 to 20. Semantic fluency was measured at each of waves 2, 3, 4 and 5. Participants were asked to name as many animals as they could in a 1-min interval, with the total number of animals named representing a continuous score of verbal fluency. Items were scored as correct if they belonged to the ‘animals’ category and were not repetitions. Tests of semantic fluency require efficient executive function with increased control of language, retrieval ability, attention and demands on frontal structures.^29^ An increase in either memory or verbal fluency scores represents a higher level of cognitive functioning.

### Loneliness

Loneliness was assessed with the abridged version of the Revised UCLA Loneliness Scale. This has been widely used as a measure of loneliness^30^ and shown to be internally reliable in measuring loneliness (α  =  0.72).^23^ The responses to these questions were coded 1 for ‘hardly ever’, 2 for ‘some of the time’ and 3 for ‘often’. The loneliness score is simply the sum of the scores of different questions, which leads to a possible score ranging from 3 to 9. The continuous score of loneliness was used in this analysis. An increase in this score represents a higher level of loneliness.

### Covariates

Information on age and sex was collected at wave 2 in 2004–05. Participants were asked for information about the highest qualifications obtained at baseline. Educational attainment was classified as low (compulsory schooling), medium (up to high school diploma) and high (university degree or higher). Wealth was calculated at baseline based on the total value of the participant’s home, financial assets and physical wealth.^26^ All respondents answered with yes or no as to whether they had any illness or disability that impaired their everyday life over an extended period; this is a standard measure of health status among older people.^31^ For depression, we used a combined algorithm of physician diagnosis and a positive score (≥3) on the seven items of the Center for Epidemiological Studies-Depression (CES-D) scale,^32^ after excluding the loneliness item from the standard eight-item CES-D. The CES-D scale has been well validated in previous studies, with a Cronbach’s alpha of 0.68.^33^^,^^34^

### Statistical analysis

**Figure 2.**
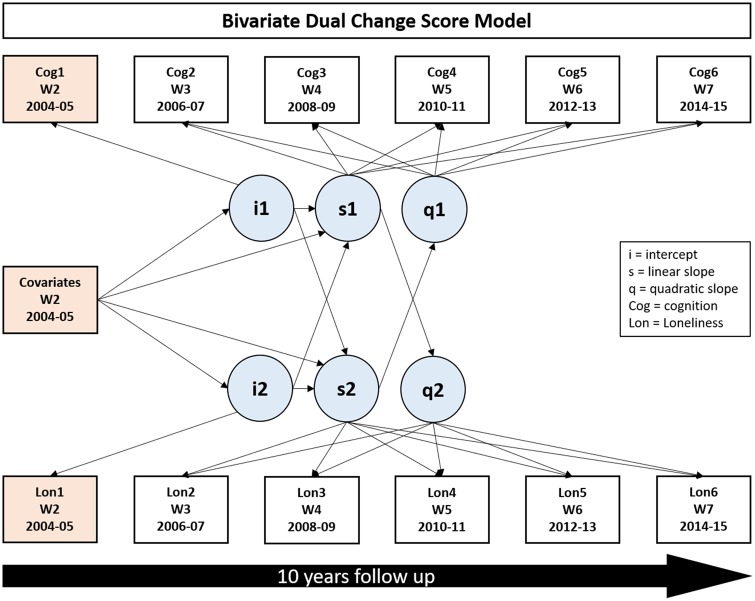
Conceptual map of analyses employed to investigate the bidirectional association between loneliness and cognitive functioning over time.

A set of bivariate dual change score models^35^^,^^36^ was fitted to examine the cross-sectional association between the level of loneliness and cognitive function at baseline, as well as the dual parallel changes in cognitive function and loneliness over the 10-year follow-up (the parallel changes in outcomes—linear or non-linear slopes). Two main models were fitted for each of the two domains of cognitive function (memory and verbal fluency; see Figure 2). The time in the study indicates the time of follow-up since baseline (in years), presenting the changes in cognitive function and loneliness between waves per every 2 years during the follow-up (see Supplementary Figure S1, available as Supplementary data at *IJE* online). In each of these models, we controlled for age, sex, education, wealth, limiting long-standing illness and depressive symptoms. The follow-up period was up to 10 years for the investigation of loneliness and memory and slightly shorter (up to 6 years) for the association with verbal fluency, as this test was not administered in ELSA at wave 6 (2012–13). For the purpose of interpretation, age was centred at 65 years based on the mean age of the sample (65.29 years).

Bayesian Information Criteria (BIC), an index that combines model parsimony and goodness of fit, was used for model selection, and based on this we selected to report the current results indicating a quadratic function of change. The outputs of these models represent the following:

- the value of the predicted intercept, linear and quadratic slope for both loneliness and cognitive function scores;
- the correlation between the initial level of the outcome and the rate of change in the outcome (e.g. individuals who start with a higher loneliness score show a steeper increase in loneliness over time, and individuals who start with poorer memory/verbal fluency show steeper decline in memory or verbal fluency performance over time);
- the role of baseline covariates on the intercept and linear slope of each outcome;
- cross-sectional associations between loneliness and each cognitive domain at baseline;
- the prospective association of baseline loneliness with changes in each cognitive domain; and the prospective association of each baseline cognitive domain with changes in loneliness;
- bivariate dual change parallel associations between the linear slope in loneliness and accelerated changes (quadratic slopes) in each cognitive domain, and between the linear slope in each cognitive domain and quadratic slope of loneliness.

This dual process modelling employs a maximum likelihood robust (MLR) estimation, which was used for all the models and is considered to produce unbiased estimates under the missing at random (MAR) assumption.^37^

Two sensitivity analyses were conducted. In the first, loneliness was dichotomized into high versus low (the reference group) using a threshold of 6 on the UCLA score (max score 9). This sensitivity analysis was conducted given the restricted variability in the range of scores for loneliness. In the second analysis, we excluded those with the lowest quintile of cognition, used in this context as a potential marker of mild cognitive impairment, and reassessed whether the relationship with loneliness persisted or was driven by those with low cognition. All data analyses were conducted using Mplus (version 6.11), Computer Software Los Angeles, CA.^38^

## Results

### Descriptive characteristics

As presented in Table 1, there were 5885 eligible participants at baseline. Participants who were not included in this analysis were older, more likely to be men, less affluent and had lower educational attainment and more limiting long-standing illness, stroke or depressive symptoms.

**Table 1.** Psychosocial and demographic characteristics of the sample at each wave of the English Longitudinal Study of Ageing (waves 2 to 7))

Variables	Wave 2	Wave 3	Wave 4	Wave 5	Wave 6	Wave 7
	(2004-05)	(2006-07)	(2008-09)	(2010-11)	(2012-13)	(2014-15)
Subjects, *n*	5885	5644	5048	4774	4417	3900
Memory						
Mean (SD)	10.5 (3.2)	10.5 (3.4)	10.4 (3.4)	10.4 (3.5)	10.5 (3.6)	10.1 (3.7)
Range	0 to 20	0 to 20	0 to 20	0 to 20	0 to 20	0 to 20
Verbal fluency						
Mean (SD)	20.8 (6.2)	20.4 (6.5)	20.8 (6.7)	20.8 (6.6)	–	–
Range	0 to 63	0 to 56	0 to 54	0 to 51	–	–
Loneliness						
Mean (SD)	4.06 (1.47)	4.14 (1.51)	4.15 (1.53)	4.12 (1.51)	4.16 (1.54)	3.98 (1.42)
Range	3 to 9	3 to 9	3 to 9	3 to 9	3 to 9	3 to 9
Age, years						
Mean (SD)	65.3 (9.0)	67.4 (9.0)	68.9 (8.7)	70.4 (8.4)	72.0 (8.2)	73.4 (7.7)
Sex, *n* (%)						
Male	2734 (44.6)	2618 (44.5)	2315 (44.2)	2165 (43.8)	1992 (43.6)	1746 (43.5)
Female	3401 (55.4)	3268 (55.5)	2925 (55.8)	2782 (56.2)	2574 (56.4)	2270 (56.5)
Education, *n* (%)						
High	1649 (26.9)	1595 (27.1)	1450 (27.7)	1421 (28.7)	1342 (29.4)	1216 (30.2)
Medium	2378 (38.8)	2288 (38.9)	2071 (39.5)	1963 (39.7)	1823 (39.9)	1641 (40.9)
Low	2108 (34.3)	2003 (34.0)	1719 (32.8)	1563 (31.6)	1401 (30.7)	1159 (28.9)
Wealth, *n* (%)						
High	2044 (33.3)	1962 (33.3)	1793 (34.2)	1762 (35.6)	1692 (37.1)	1552 (38.6)
Medium	2045 (33.3)	1940 (33.0)	1738 (33.2)	1628 (32.9)	1505 (33.0)	1316 (32.8)
Low	2046 (33.4)	1984 (33.7)	1709 (32.6)	1557 (31.5)	1369 (30.0)	1148 (28.6)
Limiting long-standing illness, *n* (%)						
No	4136 (67.4)	3975 (67.5)	3595 (68.6)	3436 (69.5)	3236 (70.9)	2893 (72.0)
Yes	1999 (32.6)	1911 (32.5)	1645 (31.4)	1511 (30.5)	1330 (29.1)	1123 (28.0)
Depressive symptoms, *n* (%)						
No	5156 (87.6)	4949 (87.7)	4427 (87.7)	4193 (87.8)	3891 (88.1)	3444 (88.3)
Yes	729 (12.4)	96 (12.3)	621 (12.3)	581 (12.2)	526 (11.9)	456 (11.7)

At baseline, the respondents had a mean [standard deviation (SD)] age of 65.3 (9.0) years, and 44.6% were men. A total of 26.9% of participants had high-level education and more than a third reported limiting longstanding illness. About 12% of participants had depressive symptoms and/or diagnosed depression. During follow-up, women and people who were older, less wealthy, had higher education and reported longstanding illness were more likely to leave the study. The average memory score decreased slightly from 10.5 at baseline (wave 1) to 10.1 at wave 7. The attrition was about 17% during the 10-year follow-up.

### Cognitive function as the outcome

As shown in Table 2 (first outcome), participants had an average memory score of *β intercept * = * *10.3, standard error (SE ) =  0.12, *P* ≤ 0.001 at baseline, and experienced a non-linear decline in memory with *β linear slope * = * *0.26, SE  =  0.05, *P* ≤ 0.001and *β quadratic slope* = −0.04, SE  =  0.01, *P* ≤ 0.001 per every 2 years. Baseline loneliness was negatively associated with baseline memory (*β intercept* = −0.03, SE  =  0.01, *P* = 0.016) and with the linear slope of change in memory (*β linear slope* = −0.07, SE  =  0.1, *P* ≤ 0.001). This suggests that higher levels of loneliness were associated with lower memory scores at baseline and with a steeper memory decline over time. Among covariates, baseline age, lower levels of education and wealth, being female and having limiting long-standing illness or depressive symptoms were related to poorer performance in memory at baseline. Older age and the lowest level of wealth also predicted a faster decline in verbal memory.

**Table 2.** Bivariate dual change score model with bidirectional coupling parameters, outcome cognition (*n*  =  5885)

	Outcome: memory			Outcome: verbal fluency		
	Exposure: loneliness					
Initial status: cognition	β	SE	*P*-value	β	SE	*P*-value
Baseline cognition (intercept i1)	10.3	0.12	≤0.001	23.58	0.25	≤0.001
Baseline loneliness (i2)	−0.03	0.01	0.016	−0.01	0.01	0.027
Baseline age	−0.11	0.01	≤0.001	−0.15	0.01	≤0.001
Sex (female vs male)	1.02	0.07	≤0.001	−0.02	0.14	0.886
Education						
Medium vs high education	−0.78	0.08	≤0.001	−1.95	0.18	≤0.001
Low vs high education	−1.96	0.09	≤0.001	−3.73	0.19	≤0.001
Wealth						
Medium vs high wealth	−0.23	0.08	0.003	−0.67	0.17	≤0.001
Low vs high wealth	−0.46	0.09	≤0.001	−0.83	0.19	≤0.001
Limiting long-standing illness	−0.28	0.07	≤0.001	−0.47	0.15	0.002
Depressive symptoms	−0.41	0.11	≤0.001	−0.76	0.21	≤0.001
**The rate of change in cognition**						
Linear slope of cognition (s1)	0.26	0.05	≤0.001	0.17	0.14	0.224
Baseline loneliness (i2)	−0.07	0.01	≤0.001	−0.09	0.03	0.003
Baseline age	−0.02	0.01	≤0.001	−0.01	0.01	0.036
Sex (female vs male)	0.02	0.02	0.176	0.02	0.03	0.598
Education						
Medium vs high education	−0.01	0.02	0.498	0.03	0.04	0.480
Low vs high education	0.01	0.02	0.511	0.02	0.04	0.666
Wealth						
Medium vs high wealth	−0.04	0.02	0.057	−0.03	0.04	0.399
Low vs high wealth	−0.04	0.02	0.037	−0.01	0.04	0.800
Limiting long-standing illness	0.02	0.02	0.177	0.04	0.04	0.229
Depressive symptoms	0.05	0.03	0.117	0.11	0.07	0.113
Quadratic slope of cognition (q1)	−0.04	0.01	≤0.001	0.04	0.03	0.250
Linear slope of loneliness (s2)	−0.07	0.04	0.088	−0.28	0.15	0.069
Variance^a^						
In initial status (i1)	3.75	0.10	≤0.001	17.38	0.49	≤0.001
In the linear rate of change (s1)	0.04	0.01	0.005	−0.01	0.04	0.919
In the quadratic rate of change (q1)	0.01	0.01	0.014	0.09	0.02	≤0.001
**Goodness of fit**	**95% CI**					
RMSEA	0.023	0.021, 0.025		0.025	0.023, 0.028	
AIC	223980.18			213426.54		
BIC	224394.35			213827.35		

***β***, beta coefficient; SE, standard error; RMSEA, root mean square error of approximation; AIC, Akaike’s Information Criterion; BIC, Bayesian Information Criterion; 95% CI, confidence intervals.

a The within-person variance is the overall residual variance in cognition (memory or verbal fluency) that is not explained by the model. The initial status variance component is the variance of individual’s intercepts about the intercept of the average person. Likewise, the rate of change variance component is the variance of individual slopes about the slope of the average person.

Participants had an average verbal fluency score of *β intercept * = * *23.58, SE  =  0.25, *P* ≤ 0.001 at baseline (see Table 2, second outcome), whereas the average linear or non-linear changes in verbal fluency were not evident. Baseline loneliness was negatively associated with baseline verbal fluency (*β intercept* = −0.01, SE  =  001, *P*   =  0.027) and linear slope of change in score of verbal fluency (*β linear slope* = −0.09, SE  =  0.03, *P* =  0.003). This indicates that participants with greater loneliness at baseline also had poorer verbal fluency at baseline and a steeper decline in verbal fluency scores over time. Baseline age, lower levels of education or wealth, having limiting long-standing illness or having depressive symptoms were related to poorer performance in verbal fluency at baseline. Older age seemed to be the only factor that was also associated with a steeper change in verbal fluency. Investigating the parallel change in cognition and loneliness, the linear slope of change in loneliness did not influence the non-linear pattern of change in either memory or verbal fluency over time.

### Loneliness as the outcome

Exploring the relationship between baseline memory and loneliness trajectory (see Table 3, first exposure), the participants in this study had a baseline score of loneliness of *β intercept * = * *3.55, SE  =  0.12, *P* ≤ 0.001, a linear slope of *β linear slope * = * *0.16, SE  =  0.04, *P* ≤ 0.001 and a quadratic slope of *β quadratic slope* = −0.02, SE  =  0.01, *P* ≤ 0.001, indicating an acceleration in the linear change over time. As previously noted, memory was found to be inversely related to the levels of loneliness at baseline and also predictive of the linear change in loneliness over time (*β linear slope* = −0.01, SE  =  0.01, *P* ≤ 0.001). This suggests that individuals with better memory scores at baseline would report a slower increase in loneliness during follow-up. Furthermore, investigating the dual changes, the linear slope in memory over time was positively related to the quadratic slope of change in loneliness (*β quadratic slope * = * *0.13, SE  =  0.03, *P*  ≤ 0.001).

**Table 3.** Bivariate dual change score model with bidirectional coupling parameters, outcome loneliness (*n*  =  5885)

	Outcome: Loneliness						
	Exposure: Memory			Exposure: Verbal fluency		
Initial status: loneliness	β	SE	*P*-value	β	SE	*P*-value
Baseline loneliness (intercept i2)	3.55	0.12	≤0.001	3.53	0.13	≤0.001
Baseline memory (i1)	−0.03	0.01	0.016	–	–	–
Baseline verbal fluency	–	–	–	−0.01	0.01	0.027
Baseline age	−0.01	0.01	≤0.001	−0.01	0.01	≤0.001
Sex (female vs male)	0.16	0.04	≤0.001	0.13	0.03	≤0.001
Education						
Medium vs high education	0.04	0.04	0.291	0.05	0.04	0.185
Low vs high education	0.11	0.05	0.026	0.12	0.05	0.016
Wealth						
Medium vs high wealth	0.13	0.04	0.001	0.13	0.04	≤0.001
Low vs high wealth	0.49	0.05	≤0.001	0.49	0.05	≤0.001
Limiting long-standing illness	0.33	0.04	≤0.001	0.34	0.04	≤0.001
Depressive symptoms	1.35	0.07	≤0.001	1.35	0.07	≤0.001
**The rate of change in loneliness**						
Linear slope of loneliness (s2)	0.16	0.04	≤0.001	0.08	0.05	0.08
Baseline memory (i1)	−0.01	0.01	0.004	–	–	–
Baseline verbal fluency (i1)	–	–	–	0.01	0.01	0.782
Baseline age	0.01	0.01	≤0.001	0.01	0.01	≤0.001
Sex (female vs male)	0.01	0.01	0.073	0.01	0.02	0.843
Education						
Medium vs high education	0.03	0.01	0.073	0.01	0.02	0.604
Low vs high education	0.01	0.02	0.760	0.02	0.03	0.364
Wealth						
Medium vs high wealth	0.04	0.01	0.009	0.04	0.02	0.098
Low vs high wealth	0.01	0.02	0.705	−0.01	0.03	0.893
Limiting longstanding illness	0.01	0.01	0.478	0.01	0.02	0.834
Depressive symptoms	−0.09	0.02	≤0.001	−0.10	0.04	0.007
Quadratic slope of loneliness (q2)	−0.02	0.01	≤0.001	0.01	0.01	0.966
Linear change in memory (s1)	0.13	0.03	≤0.001	–	–	–
Linear change in verbal fluency (s1)	–	–	–	0.12	0.05	0.010
Variance^a^						
In initial status	1.15	0.03	≤0.001	1.13	0.03	≤0.001
In the linear rate of change	0.03	0.01	≤0.001	0.04	0.01	≤0.001
In the quadratic rate of change	0.01	0.01	0.010	0.01	0.01	0.730
**Goodness of fit**	**95% CI**						
RMSEA	0.023	0.021, 0.026		0.025	0.023, 0.028	
AIC	223983.85			213426.54		
BIC	224404.70			213827.35		

***β***, beta coefficient; SE, standard error; RMSEA, root mean square error of approximation; AIC, Akaike’s Information Criterion; BIC, Bayesian Information Criterion; 95% CI, confidence intervals.

a The within-person variance is the overall residual variance in loneliness that is not explained by the model. The initial status variance component is the variance of individual’s intercepts about the intercept of the average person. Likewise, the rate of change variance component is the variance of individual slopes about the slope of the average person.

Greater age at baseline was negatively associated with baseline level of loneliness but positively associated with a greater linear increase in loneliness over time. Being female, having lower levels of education or wealth, having limiting long-standing illness and having depressive symptoms were associated with a higher level of baseline loneliness. Greater baseline age, a medium compared with a higher level of wealth and having depressive symptoms predicted a faster increase in loneliness over time.

In the model using baseline verbal fluency as a predictor of loneliness (see Table 3, second exposure), we noticed an inverse cross-sectional association suggesting that higher baseline scores of verbal fluency were associated with less loneliness at baseline. However, we did not observe a prospective association between loneliness at baseline and change in verbal fluency over time. Investigating the dual changes, we found that the linear slope in verbal fluency was positively related to the quadratic slope of change in loneliness (*β quadratic slope * = * *0.12, SE  =  0.05, *P* =  0.01). The relationships of covariates with loneliness followed similar patterns to those in the model using memory as a predictor.

### Sensitivity analyses

The results of the first sensitivity analysis, in which loneliness was dichotomised into low versus high, using a threshold of 6 on the UCLA score (see Supplementary Tables S1 and S2, available as Supplementary data at *IJE* online), showed a similar pattern, highlighting a cross-sectional association between high loneliness and cognitive functioning and a marginal prospective association between high loneliness at baseline and a steeper decline in memory. An increase in loneliness over time also predicted an acceleration in the verbal fluency decline over time. Furthermore, the level of verbal fluency at baseline predicted a greater increase in loneliness over time, supporting the initial findings from our main analyses and the potential bidirectionality at play.

The results of the second sensitivity analysis are from an analytical sample from which we excluded those with the lowest level of cognitive functioning at baseline (*n * =  3606, see Tables S3 and S4, available as Supplementary data at *IJE* online). The results highlight a lack of cross-sectional association between loneliness and cognitive functioning at baseline in this restricted analytical sample of individuals who were cognitively fit at baseline, but still found a bidirectional association between baseline loneliness and a steeper decline in either memory or verbal fluency over time, as well as between better baseline memory and a slower increase in loneliness over time. Last, these results show a similar pattern of dual changes over time, with the linear rates of change in either memory or verbal fluency being predictive of accelerated changes in loneliness.

## Discussion

In a nationally representative sample of the English population aged 50 years or older, we found evidence of a bidirectional association between loneliness and cognitive function, as well as some evidence of a dual process of change in these factors. Greater loneliness at baseline was associated with a more rapid decline in both memory and verbal fluency. Moreover, better memory at baseline was linked to a slower worsening in loneliness over a 10-year follow-up, independent of age, sex, education, wealth, limiting long-standing illness and depressive symptoms. However, this was not the case for baseline verbal fluency in predicting a change in loneliness levels.

Interestingly, the independent rate of decline in both memory and verbal fluency was associated with an accelerated change in loneliness whereas, in reverse, the linear slope of loneliness did not predict an acceleration in cognitive decline over time. Therefore, the current findings highlight a bidirectional association between baseline levels of cognition and loneliness, as well as between baseline loneliness and linear changes in each of the two cognitive domains (memory and verbal fluency) over time. However, when examining the dual process of change, only a change in cognition was associated with an acceleration in loneliness, but an increase in loneliness was not predictive of an acceleration in cognitive functioning decline. However, we cannot preclude that a more prominent decline would not happen during a longer period of follow-up.

Successful performance on verbal fluency testing requires not only executive control but also active maintenance of vocabulary knowledge, semantic, phonemic and lexical fluency, and social and mental processing. Many aspects of cognitive functioning—primarily those associated with executive processing and other functions of the frontal lobe—do appear to deteriorate with age, but this is not the case for all ageing individuals, many of whom may be able to maintain or even improve their cognitive performance with age. Age-related changes in memory and other cognitive abilities occur at different rates; for example, reasoning skills, visuospatial facility and verbal memory decline more rapidly over the life course,^39^^,^^40^ whereas vocabulary, calculation and decision making are more resistant to ageing.^41^ Investigating cognitive decline in its most fluid abilities (memory and verbal fluency) and their determinants is important because early detection of severe levels of cognitive decline could be targeted for special monitoring of the stages of progression from ‘normal ageing’ to the subtle signs of subclinical neurodegenerative disease, which can precede dementia diagnosis by many years. Our observations are in line with previous studies^5–9^ that showed that cognitive abilities like verbal knowledge and access to lexicon do not decline substantially over time.

Some of our findings are in line with the current evidence. For example, in a population-based study of older Finnish adults, loneliness was related to cognitive decline at 10-year follow-up but not to earlier periods.^19^ Unlike memory, the scores for verbal fluency in our study were available up to wave 5 only (6-year follow-up). Scores from later waves may be necessary to observe a clearer trend of change in verbal fluency over time in relation to baseline loneliness. Education had the largest effects on baseline levels of memory and verbal fluency. Consistent with previous findings, depressive symptoms were negatively and cross-sectionally related to both domains of cognition investigated here (memory and verbal fluency), as well as to baseline levels of loneliness. However, baseline depressive symptoms did not predict the rate of decline in either memory or verbal fluency during the follow-up period in our analysis, which is in contrast to previous evidence highlighting that baseline depressive symptoms predicted a steeper decline in executive and global cognitive function in men.^42^ It is important to note that our analyses reveal a role of loneliness independent from overall depressive symptoms in the prospective association with cognitive decline over almost a decade of follow-up.

Our study is one of very few to examine a bidirectional association between cognitive function and loneliness, as well as the dual parallel changes between these factors. Both memory and verbal fluency were inversely associated with loneliness at baseline and were predictive of changes in loneliness and accelerations in these changes over time. However, a change in loneliness did not predict an acceleration in the rates of cognitive decline observed in these analyses. This is somehow consistent with previous findings. Data from HRS have been used to explore the bidirectional association between loneliness and cognitive function in a study conducted by Donovan and colleagues,^21^ where greater loneliness at baseline predicted a more rapid cognitive decline over 12 years (from 1998 to 2010), independent of sociodemographic factors, social networks and physical health. However, in their study, this association was attenuated once depressive symptoms were taken into account, indicating that the effects of loneliness and depressive symptoms may not always be distinct.

Nevertheless, examining the bidirectionality of this association within the same study, the global levels of cognitive function at baseline were not a strong predictor of loneliness. The difference is probably related to the use of global cognition instead of specific cognitive domains. Furthermore, they assessed loneliness using only one question from the eight-item version of the Centre for Epidemiologic Studies Depression Scale, which may be less sensitive than the UCLA Loneliness Scale (used in our study), and therefore this may have also led to an underestimation of the bidirectional associations between loneliness and global cognitive function that they found. Last, we were interested in examining memory and verbal fluency separately, to help unmask the underlying differences among different domains of cognition and their predicting role in relation to a change in loneliness.

## Biological mechanisms

Biologically, loneliness is proposed as a risk factor for chronic inflammation, immune system impairment and activation of the hypothalamic–pituitary–adrenal (HPA) axis, which could subsequently lead to a decrease in dendritic arborisation in the hippocampus and prefrontal cortex.^43^ Ultimately, these changes could result in a faster neurodegeneration process, with ageing contributing to cognitive dysfunction.^44^ Besides, the reduced capability of self-regulation is one of the consequences of loneliness, leading to an increased risk in adopting unhealthy lifestyle behaviours such as drinking and smoking, which will in turn impair cognitive performance with age.^44^ On the other hand, loneliness could also be considered as a behavioural response to a potential deterioration in cognitive functioning over time or risk of cognitive impairment or dementia onset. Memory loss is often characterised as becoming forgetful and disorganised, which may be an early sign of cognitive dysfunction in elderly individuals without dementia.^45^^,^^46^ This, in turn, may also lead to isolation and loneliness due to the stigma of cognitive decline or impairment.^47^

## Strengths and limitations

This work has several strengths and limitations. First, in this sample, 17% of core participants were lost to attrition during follow-up, and those who dropped out were older males, less educated, less affluent and with long-standing limiting illness: indicators that were associated with a poorer cognitive function. Therefore these results may be conservative, but it is reasonable to assume that the associations found could have been stronger if they were not lost to follow-up.^21^ Furthermore, the statistical models employed used maximum likelihood estimation, and model assumptions were verified by examining residuals computed from the predicted values.^37^ Second, loneliness was measured using the revised UCLA Loneliness Scale, which refers more to social connections rather than to an objective feeling of loneliness.^48^ Although the UCLA Loneliness Scale has been shown to be consistent with other instruments,^49^ it is still difficult to know whether it is in fact loneliness that was measured. Furthermore, individuals may not necessarily admit that they are lonely due to the associated social stigma,^12^ and this potential self-reporting bias may have resulted in a slight underestimation of a feeling of loneliness. Moreover, the unbalanced sensitivity of measurements of loneliness may result in a slight underestimation of this bidirectional relationship with cognitive functioning. Regarding cognitive functioning, we benefited from having two repeated measures of memory and verbal fluency, but an additional instrument for investigating executive functioning would have been desirable in this study.

Nevertheless, the study benefits from several strengths. These are the use of a representative sample of the English population in their mid and later life, repeated measures of loneliness and cognitive functioning over a long period of follow-up, and the use of complex modelling to explore the bidirectional relationships between loneliness and cognitive function using independent tests of memory and verbal fluency. To our knowledge, this is the first study to explore the dual parallel changes in loneliness and cognitive function over almost a decade.

In conclusion, loneliness appears to be associated with poorer cognitive function at baseline on both measures of memory and verbal fluency, as well as contributing to a worsening in memory and verbal fluency over time. A bidirectional association was only found for baseline memory and not for verbal fluency, predicting subsequent changes in loneliness over time. Exploring the parallel changes in loneliness and cognitive functioning, we found that linear slopes of decline in either memory or verbal fluency predicted an acceleration in loneliness over time, whereas the linear rate of change in loneliness did not predict an accelerated change in cognitive functioning. The interlinkage of loneliness and subsequent cognitive decline is noteworthy and may have public health implications, raising the possibility that initiatives aimed at reducing loneliness among older people may impact on cognitive resilience in later life.

## Supplementary Material

dyz085_Supplementary_Data
