# Supplementary material for: Exploring the bidirectional associations between loneliness and cognitive functioning over 10 years: the English longitudinal study of ageing
Source: Int J Epidemiol. 2019 May 5;48(6):1937–48. doi: 10.1093/ije/dyz085 (PMC6929532; doi:10.1093/ije/dyz085)
Supplement: dyz085_Supplementary_Data [file dyz085_supplementary_data.zip › dyz085-suppl_data/Supplementary Data.docx]

**Sensitivity analysis I: Using a dichotomised loneliness score instead of a continuous score**

A similar set of bivariate dual change score models were fitted for each of the two domains of cognitive function (memory and verbal fluency), in relation to loneliness (used as a categorical outcome). In the parameterisation of the growth model used in these analyses, the thresholds of the outcome variable at the 6-time points are held equal as the default in MPlus. Therefore, the mean of the intercept growth factor is fixed at zero. The mean of the slope growth factor and the variances of the intercept and slope growth factors are estimated as the default, and the growth factor of the bivariate dual change score model’s covariance is estimated as the default because the growth factors are independent (exogenous) variables.

Table S1. Bivariate Dual Change Score Model with Bidirectional Coupling Parameters, using a dichotomised score of loneliness - outcome cognition (N=5,885)

|  | **Outcome: Memory** | | | | | **Outcome: Verbal fluency** | | | | |
| --- | --- | --- | --- | --- | --- | --- | --- | --- | --- | --- |
|  | **Exposure: Loneliness (high vs low)** | | | | | | | | | |
| **Initial status: Cognition** | ***β*** | ***SE*** | | ***p-value*** | | ***β*** | ***SE*** | | ***p-value*** | |
| Baseline cognition (intercept i1) | 10.29 | 0.12 | | p≤0.001 | | 23.78 | 0.27 | | p≤0.001 | |
| Baseline loneliness (high vs. low) (i2) | -0.14 | 0.04 | | p≤0.001 | | -0.07 | 0.02 | | p≤0.001 | |
| Baseline age | -0.10 | 0.01 | | p≤0.001 | | -0.14 | 0.01 | | p≤0.001 | |
| Sex (female vs. male) | 0.99 | 0.07 | | p≤0.001 | | -0.15 | 0.15 | | 0.314 | |
| Education |  |  | |  | |  |  | |  | |
| Medium vs. high education | -0.78 | 0.08 | | p≤0.001 | | -1.96 | 0.19 | | p≤0.001 | |
| Low vs. high education | -1.96 | 0.09 | | p≤0.001 | | -3.69 | 0.20 | | p≤0.001 | |
| Wealth |  |  | |  | |  |  | |  | |
| Medium vs. high wealth | -0.24 | 0.08 | | 0.004 | | -0.72 | 0.18 | | p≤0.001 | |
| Low vs. high wealth | -0.46 | 0.09 | | p≤0.001 | | -0.88 | 0.20 | | p≤0.001 | |
| Limiting longstanding illness | -0.28 | 0.08 | | p≤0.001 | | -0.54 | 0.16 | | 0.001 | |
| Depressive symptoms | -0.36 | 0.11 | | 0.001 | | -0.41 | 0.22 | | 0.066 | |
| **The rate of change in cognition** |  | | | | |  | | | | |
| Linear slope of cognition (s1) | 0.11 | 0.04 | | 0.785 | | -0.39 | 0.17 | | 0.024 | |
| Baseline loneliness (high vs. low) (i2) | -0.01 | 0.01 | | 0.050 | | 0.01 | 0.02 | | 0.444 | |
| Baseline age | -0.02 | 0.01 | | p≤0.001 | | -0.02 | 0.01 | | p≤0.001 | |
| Sex (female vs. male) | 0.03 | 0.02 | | 0.115 | | 0.17 | 0.08 | | 0.056 | |
| Education |  |  | |  | |  |  | |  | |
| Medium vs. high education | -0.03 | 0.02 | | 0.237 | | 0.05 | 0.11 | | 0.636 | |
| Low vs. high education | 0.01 | 0.03 | | 0.913 | | 0.01 | 0.12 | | 0.960 | |
| Wealth |  |  | |  | |  |  | |  | |
| Medium vs. high wealth | -0.05 | 0.03 | | 0.073 | | 0.04 | 0.10 | | 0.714 | |
| Low vs. high wealth | -0.07 | 0.03 | | 0.014 | | -0.09 | 0.12 | | 0.443 | |
| Limiting longstanding illness | 0.01 | 0.02 | | 0.532 | | 0.09 | 0.09 | | 0.325 | |
| Depressive symptoms | -0.02 | 0.04 | | 0.638 | | -0.47 | 0.22 | | 0.031 | |
| Quadratic slope of cognition (q1) | -0.04 | 0.01 | | p≤0.001 | | 0.09 | 0.05 | | 0.053 | |
| Linear slope of loneliness (s2) | 0.04 | 0.07 | | 0.499 | | -0.29 | 0.12 | | 0.011 | |
| Variance ^a^ |  | |  | |  |  | |  | |  |
| In initial status | 3.72 | | 0.10 | | p≤0.001 | 17.73 | | 0.53 | | p≤0.001 |
| In the linear rate of change | 0.07 | | 0.02 | | p≤0.001 | -0.02 | | 0.23 | | 0.935 |
| In the quadratic rate of change | 0.01 | | 0.01 | | 0.884 | 0.09 | | 0.04 | | 0.022 |
| Goodness of fit |  | | | | |  | | | | |
| AIC | 158717.46 | | | | | 148172.48 | | | | |
| BIC | 159091.55 | | | | | 148533.21 | | | | |

***β***, beta coefficient; SE, standard error; RMSEA, Root Mean Square Error of Approximation; AIC, Akaike’s Information Criterion; BIC, Bayesian Information Criterion; 95% CI, Confidence Intervals

^a^ The within-person variance is the overall residual variance in cognition (memory or verbal fluency) that is not explained by the model. The initial status variance component is the variance of individuals’ intercepts about the intercept of the average person. Likewise, the rate of change variance component is the variance of individual slopes about the slope of the average person.

Table S2. Bivariate Dual Change Score Model with Bidirectional Coupling Parameters, using a dichotomised score of loneliness as the outcome (N=5,885)

|  | **Outcome: Loneliness (high** **vs low)** | | | | | | | | | | |
| --- | --- | --- | --- | --- | --- | --- | --- | --- | --- | --- | --- |
|  | **Exposure: Memory** | | | | | | **Exposure: Verbal fluency** | | | | |
| **Initial status: Loneliness (intercept i2)** | ***β*** | | ***SE*** | | ***p-value*** | | ***β*** | ***SE*** | | ***p-value*** | |
| Baseline memory (i1) | -0.14 | | 0.04 | | p≤0.001 | | - | - | | - | |
| Baseline verbal fluency (i1) | - | | - | | - | | -0.07 | 0.02 | | p≤0.001 | |
| Baseline age | -0.03 | | 0.01 | | 0.003 | | -0.01 | 0.01 | | 0.067 | |
| Sex (female vs. male) | 0.56 | | 0.12 | | p≤0.001 | | 0.38 | 0.12 | | 0.002 | |
| Education |  | |  | |  | |  |  | |  | |
| Medium vs. high education | 0.15 | | 0.15 | | 0.315 | | 0.14 | 0.16 | | 0.395 | |
| Low vs. high education | 0.14 | | 0.17 | | 0.426 | | 0.19 | 0.18 | | 0.281 | |
| Wealth |  | |  | |  | |  |  | |  | |
| Medium vs. high wealth | 0.27 | | 0.15 | | 0.073 | | 0.27 | 0.16 | | 0.084 | |
| Low vs. high wealth | 1.36 | | 0.16 | | p≤0.001 | | 1.40 | 0.16 | | p≤0.001 | |
| Limiting longstanding illness | 0.87 | | 0.12 | | p≤0.001 | | 0.87 | 0.13 | | p≤0.001 | |
| Depressive symptoms | 2.78 | | 0.16 | | p≤0.001 | | 2.90 | 0.18 | | p≤0.001 | |
| **The rate of change in loneliness** |  | | | | | |  | | | | |
| Linear slope of loneliness (s2) | 0.21 | | 0.15 | | 0.155 | | -0.14 | 0.16 | | 0.381 | |
| Baseline memory (i1) | -0.01 | | 0.01 | | 0.675 | | - | - | | - | |
| Baseline verbal fluency (i1) | - | | - | | - | | 0.01 | 0.01 | | 0.047 | |
| Baseline age | 0.01 | | 0.01 | | 0.010 | | 0.01 | 0.01 | | 0.374 | |
| Sex (female vs. male) | 0.05 | | 0.04 | | 0.251 | | 0.09 | 0.06 | | 0.096 | |
| Education |  | |  | |  | |  |  | |  | |
| Medium vs. high education | 0.04 | | 0.05 | | 0.436 | | 0.07 | 0.07 | | 0.298 | |
| Low vs. high education | 0.08 | | 0.06 | | 0.143 | | 0.13 | 0.08 | | 0.092 | |
| Wealth |  | |  | |  | |  |  | |  | |
| Medium vs. high wealth | 0.11 | | 0.05 | | 0.024 | | 0.12 | 0.07 | | 0.096 | |
| Low vs. high wealth | -0.06 | | 0.05 | | 0.293 | | -0.09 | 0.07 | | 0.223 | |
| Limiting longstanding illness | 0.03 | | 0.04 | | 0.470 | | 0.06 | 0.06 | | 0.277 | |
| Depressive symptoms | -0.14 | | 0.05 | | 0.004 | | -0.29 | 0.13 | | 0.026 | |
| Quadratic slope of loneliness (q2) | -0.08 | | 0.01 | | p≤0.001 | | -0.08 | 0.01 | | p≤0.001 | |
| Linear change in memory (s1) | 0.04 | | 0.07 | | 0.499 | | - | - | | - | |
| Linear change in verbal fluency (s1) | | - | - | | - | | -0.07 | 0.03 | | 0.038 | |
| Variance ^a^ |  | | | | | |  | | | | |
| In initial status | 7.07 | | | 0.39 | | p≤0.001 | 7.21 | | 0.40 | | p≤0.001 |
| In the linear rate of change | 0.20 | | | 0.04 | | p≤0.001 | 0.19 | | 0.04 | | p≤0.001 |
| In the quadratic rate of change | 0.01 | | | 0.01 | | 0.220 | 0.01 | | 0.01 | | 0.079 |
| Goodness of fit |  | | | | | |  | | | | |
| AIC | 158717.46 | | | | | | 148172.48 | | | | |
| BIC | 159091.55 | | | | | | 148533.21 | | | | |

***β***, beta coefficient; SE, standard error; AIC, Akaike’s Information Criterion; BIC, Bayesian Information Criterion

^a^ The within-person variance is the overall residual variance in loneliness that is not explained by the model. The initial status variance component is the variance of individuals’ intercepts about the intercept of the average person. Likewise, the rate of change variance component is the variance of individual slopes about the slope of the average person.

**Sensitivity analysis II: Excluding those with low cognition at baseline**

Table S3. Bivariate Dual Change Score Model with Bidirectional Coupling Parameters, outcome cognition (N=3,606)

|  | **Outcome: Memory** | | | | | **Outcome: Verbal fluency** | | | | |
| --- | --- | --- | --- | --- | --- | --- | --- | --- | --- | --- |
|  | **Exposure: Loneliness** | | | | | | | | | |
| **Initial status: Cognition** | ***β*** | ***SE*** | | ***p-value*** | | ***β*** | ***SE*** | | ***p-value*** | |
| Baseline cognition (intercept i1) | 11.54 | 0.12 | | p≤0.001 | | 24.96 | 0.28 | | p≤0.001 | |
| Baseline loneliness (i2) | 0.02 | 0.02 | | 0.223 | | 0.01 | 0.01 | | 0.732 | |
| Baseline age | -0.06 | 0.01 | | p≤0.001 | | -0.11 | 0.01 | | p≤0.001 | |
| Sex (female vs. male) | 0.69 | 0.07 | | p≤0.001 | | -0.11 | 0.16 | | 0.481 | |
| Education |  |  | |  | |  |  | |  | |
| Medium vs. high education | -0.48 | 0.08 | | p≤0.001 | | -1.39 | 0.19 | | p≤0.001 | |
| Low vs. high education | -1.20 | 0.09 | | p≤0.001 | | -2.81 | 0.21 | | p≤0.001 | |
| Wealth |  |  | |  | |  |  | |  | |
| Medium vs. high wealth | -0.20 | 0.08 | | 0.015 | | -0.38 | 0.18 | | 0.038 | |
| Low vs. high wealth | -0.09 | 0.09 | | 0.298 | | -0.11 | 0.21 | | 0.602 | |
| Limiting longstanding illness | -0.01 | 0.08 | | 0.994 | | 0.45 | 0.18 | | 0.804 | |
| Depressive symptoms | -0.29 | 0.11 | | 0.009 | | -0.58 | 0.24 | | 0.017 | |
| **The rate of change in cognition** |  | | | | |  | | | | |
| Linear slope of cognition (s1) | -0.12 | 0.06 | | 0.053 | | -0.06 | 0.21 | | 0.768 | |
| Baseline loneliness (i2) | -0.08 | 0.01 | | p≤0.001 | | -0.17 | 0.04 | | p≤0.001 | |
| Baseline age | -0.03 | 0.01 | | p≤0.001 | | -0.03 | 0.01 | | 0.014 | |
| Sex (female vs. male) | 0.08 | 0.02 | | p≤0.001 | | -0.01 | 0.06 | | 0.828 | |
| Education |  |  | |  | |  |  | |  | |
| Medium vs. high education | -0.06 | 0.03 | | 0.035 | | -0.05 | 0.08 | | 0.504 | |
| Low vs. high education | -0.12 | 0.03 | | p≤0.001 | | -0.10 | 0.10 | | 0.317 | |
| Wealth |  |  | |  | |  |  | |  | |
| Medium vs. high wealth | -0.03 | 0.03 | | 0.282 | | -0.11 | 0.08 | | 0.188 | |
| Low vs. high wealth | -0.03 | 0.03 | | 0.334 | | -0.05 | 0.09 | | 0.595 | |
| Limiting longstanding illness | -0.05 | 0.03 | | 0.080 | | -0.08 | 0.07 | | 0.282 | |
| Depressive symptoms | 0.08 | 0.05 | | 0.071 | | 0.27 | 0.15 | | 0.063 | |
| Quadratic slope of cognition (q1) | 0.01 | 0.01 | | 0.190 | | 0.14 | 0.05 | | 0.002 | |
| Linear slope of loneliness (s2) | 0.07 | 0.04 | | 0.101 | | 0.09 | 0.24 | | 0.705 | |
| Variance ^a^ |  | |  | |  |  | |  | |  |
| In initial status | 2.19 | | 0.08 | | p≤0.001 | 12.64 | | 0.54 | | p≤0.001 |
| In the linear rate of change | 0.14 | | 0.02 | | p≤0.001 | 0.08 | | 0.13 | | 0.537 |
| In the quadratic rate of change | -0.01 | | 0.01 | | 0.009 | 0.13 | | 0.03 | | p≤0.001 |
| Goodness of fit | 95% CI | | | | |  | | | | |
| RMSEA | 0.033 | 0.031, 0.036 | | | | 0.031 | | 0.028, 0.034 | | |
| AIC | 138888.81 | | | | | 132639.89 | | | | |
| BIC | 139272.61 | | | | | 133011.32 | | | | |

***β***, beta coefficient; SE, standard error; RMSEA, Root Mean Square Error of Approximation; AIC, Akaike’s Information Criterion; BIC, Bayesian Information Criterion; 95% CI, Confidence Intervals

^a^ The within-person variance is the overall residual variance in cognition (memory or verbal fluency) that is not explained by the model. The initial status variance component is the variance of individuals’ intercepts about the intercept of the average person. Likewise, the rate of change variance component is the variance of individual slopes about the slope of the average person.

Table S4. Bivariate Dual Change Score Model with Bidirectional Coupling Parameters, outcome loneliness (N=3,606)

|  | **Outcome: Loneliness** | | | | | | | | | | | | |
| --- | --- | --- | --- | --- | --- | --- | --- | --- | --- | --- | --- | --- | --- |
|  | **Exposure:**  **Memory** | | | | | | | **Exposure: Verbal fluency** | | | | | |
| **Initial status: Loneliness** | ***β*** | | ***SE*** | | | ***p-value*** | | ***β*** | ***SE*** | | | ***p-value*** | |
| Baseline loneliness (intercept i2) | 2.98 | | 0.21 | | | p≤0.001 | | 3.20 | 0.19 | | | p≤0.001 | |
| Baseline memory (i1) | 0.02 | | 0.02 | | | 0.223 | | - | - | | | - | |
| Baseline verbal fluency (i1) | - | | - | | | - | | 0.01 | 0.01 | | | 0.732 | |
| Baseline age | -0.01 | | 0.01 | | | 0.013 | | -0.01 | 0.01 | | | 0.002 | |
| Sex (female vs. male) | 0.15 | | 0.04 | | | p≤0.001 | | 0.15 | 0.04 | | | p≤0.001 | |
| Education |  | |  | | |  | |  |  | | |  | |
| Medium vs. high education | 0.03 | | 0.05 | | | 0.550 | | 0.02 | 0.05 | | | 0.675 | |
| Low vs. high education | 0.07 | | 0.06 | | | 0.276 | | 0.05 | 0.06 | | | 0.412 | |
| Wealth |  | |  | | |  | |  |  | | |  | |
| Medium vs. high wealth | 0.16 | | 0.05 | | | 0.001 | | 0.14 | 0.05 | | | 0.002 | |
| Low vs. high wealth | 0.46 | | 0.06 | | | p≤0.001 | | 0.45 | 0.06 | | | p≤0.001 | |
| Limiting longstanding illness | 0.37 | | 0.05 | | | p≤0.001 | | 0.36 | 0.05 | | | p≤0.001 | |
| Depressive symptoms | 1.28 | | 0.07 | | | p≤0.001 | | 1.29 | 0.09 | | | p≤0.001 | |
| **The rate of change in loneliness** |  | | | | | | |  | | | | | |
| Linear slope of loneliness (s2) | 0.25 | | 0.06 | | | p≤0.001 | | 0.06 | 0.06 | | | 0.334 | |
| Baseline memory (i1) | -0.01 | | 0.01 | | | 0.005 | | - | - | | | - | |
| Baseline verbal fluency (i1) | - | | - | | | - | | 0.01 | 0.01 | | | 0.899 | |
| Baseline age | 0.01 | | 0.01 | | | p≤0.001 | | 0.01 | 0.01 | | | p≤0.001 | |
| Sex (female vs. male) | -0.01 | | 0.01 | | | 0.759 | | 0.02 | 0.02 | | | 0.438 | |
| Education |  | |  | | |  | |  |  | | |  | |
| Medium vs. high education | 0.03 | | 0.01 | | | 0.020 | | 0.04 | 0.02 | | | 0.078 | |
| Low vs. high education | 0.05 | | 0.02 | | | 0.011 | | 0.06 | 0.03 | | | 0.047 | |
| Wealth |  | |  | | |  | |  |  | | |  | |
| Medium vs. high wealth | 0.02 | | 0.01 | | | 0.101 | | 0.05 | 0.03 | | | 0.034 | |
| Low vs. high wealth | -0.02 | | 0.02 | | | 0.355 | | 0.01 | 0.03 | | | 0.900 | |
| Limiting longstanding illness | 0.02 | | 0.01 | | | 0.077 | | 0.05 | 0.03 | | | 0.082 | |
| Depressive symptoms | -0.09 | | 0.02 | | | p≤0.001 | | -0.12 | 0.04 | | | 0.006 | |
| Quadratic slope of loneliness (q2) | -0.01 | | 0.01 | | | 0.498 | | 0.03 | 0.02 | | | 0.155 | |
| Linear change in memory (s1) | 0.05 | | 0.01 | | | p≤0.001 | | - | - | | | - | |
| Linear change in verbal fluency (s1) | | - | - | | | - | | 0.06 | 0.02 | | | 0.007 | |
| Variance ^a^ |  | | | | | | |  | | | | | |
| In initial status | 0.98 | | | 0.03 | | | p≤0.001 | 1.02 | | 0.04 | | | p≤0.001 |
| In the linear rate of change | 0.02 | | | 0.01 | | | p≤0.001 | 0.03 | | 0.01 | | | p≤0.001 |
| In the quadratic rate of change | 0.01 | | | 0.01 | | | 0.220 | 0.01 | | 0.01 | | | 0.244 |
| Goodness of fit |  | | | | | | |  | | | | | |
| RMSEA | 0.033 | | | | 0.031, 0.036 | | | 0.025 | | | 0.023, 0.028 | | |
| AIC | 138888.81 | | | | | | | 213426.54 | | | | | |
| BIC | 139272.61 | | | | | | | 213827.35 | | | | | |

***β***, beta coefficient; SE, standard error; RMSEA, Root Mean Square Error of Approximation; AIC, Akaike’s Information Criterion; BIC, Bayesian Information Criterion; 95% CI, Confidence Intervals

^a^ The within-person variance is the overall residual variance in loneliness that is not explained by the model. The initial status variance component is the variance of individuals’ intercepts about the intercept of the average person. Likewise, the rate of change variance component is the variance of individual slopes about the slope of the average person.

Person i

Loneliness score 1:

wave 2

Loneliness score 2:

wave 3

Loneliness score 3:

wave 4

Loneliness score 4:

wave 5

Loneliness score 5:

wave 6

Loneliness score 6:

wave 7

Level 2: individuals

Level 1: observations

Cognition score 1:

wave 2

Cognition score 2:

wave 3

Cognition score 3:

wave 4

Cognition score 4:

wave 5

Cognition score 5:

wave 6

Cognition score 6:

wave 7

Level 1: observations

Supplement Figure S1. **The structure of analyses in the dual score model of change**
