# Supplementary figures and images for: Exploring the bidirectional associations between loneliness and cognitive functioning over 10 years: the English longitudinal study of ageing
Source: Int J Epidemiol. 2019 May 5;48(6):1937–48. doi: 10.1093/ije/dyz085 (PMC6929532; doi:10.1093/ije/dyz085)

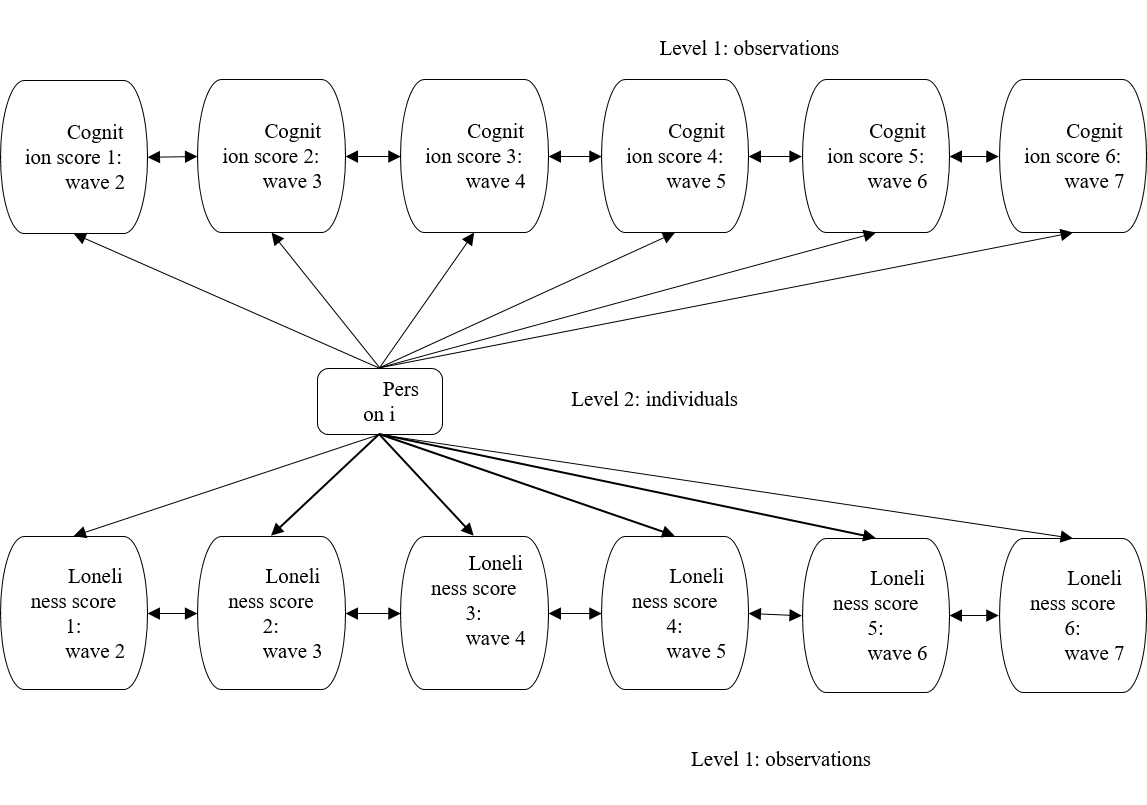

Supplement: dyz085_Supplementary_Data [file dyz085_supplementary_data.zip › dyz085-suppl_data/Supplementary Figure.tif]
